# Supplementary material for: Using Arts-Based Methodologies to Understand Adolescent and Youth Manifestations, Representations, and Potential Causes of Depression and Anxiety in Low-Income Urban Settings in Peru
Source: Int J Environ Res Public Health. 2022 Nov 23;19(23):15517. doi: 10.3390/ijerph192315517 (PMC9737895; doi:10.3390/ijerph192315517)

Supplementary File S1

Artistic outputs from adolescents

Theme: Anxiety

Similes

Participant 1

- Cuando estoy angustiada me siento como una aguja pinchando mi piel.
- Cuando estoy angustiada me siento un gato con cabeza de honguito.

Participant 2

- Cuando estoy angustiada me siento como si Dios no estuviera conmigo.
- Cuando estoy angustiada me siento como si mi grito se ahogara en mis labios.

Participant 3

- Cuando estoy angustiado me siento como un monedero vacío.
- Cuando estoy triste me siento como una clava sin alma.

Participant 4

- Cuando estoy angustiada me siento como Cubo Rubik revuelto.
- Cuando estoy angustiada me siento como una guitarra desafinada.

Images

Participant 1

Not included due to copyright restrictions. The image was of a drawing of a young clown with sleepy eyes, a birthday hat, a blush on the cheeks and the nose, and one earring.

Participant 2

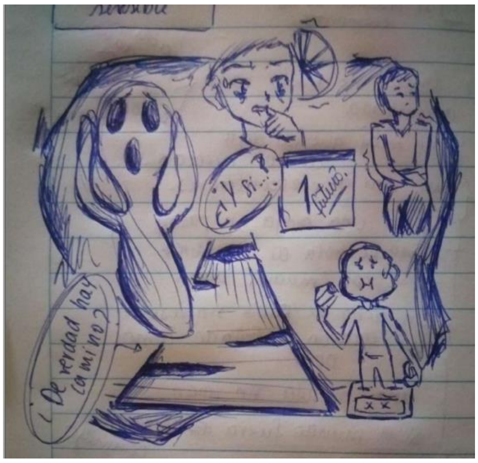

A second image, not included due to copyright restrictions, was also included. It was a photograph of Edvard Munch’s art piece, The Scream.

Participant 3

Not included due to copyright restrictions. The image was of a man lying down in bed at night with his eyes open and an alarm clock showing the time of 3:25.

Participant 4

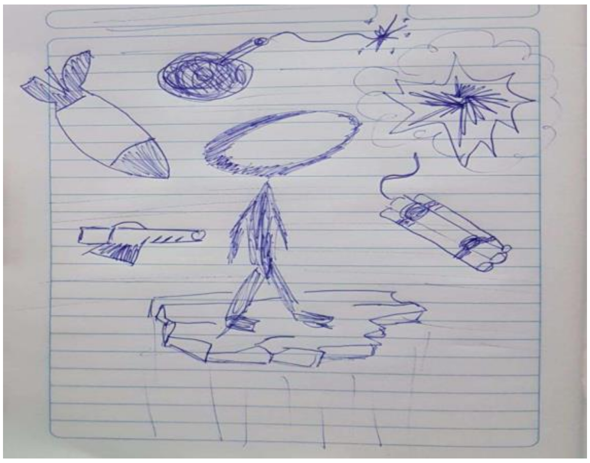

Monologues

Participant 1

Soy la ansiedad, me gusta jalarme los cabellos mientras escucho la lluvia caer contra el techo, siempre uso un gorro de cumpleaños por si alguien me invita al suyo, aunque aún así no me dejarían ir. Mis manos botan sudor y no lo puedo evitar. Por las noches no puedo dormir y eso hace que a la mañana siguiente tenga ojeras negras y profundas.

Participant 2

Buenos días.
Buenos días. O buenas tardes. ¿O noches? ¿Es ya de mañana? Cierto, ¿no? Oh, entonces estaba bien buenos días...
Soy Preocupación, mucho gusto. No. ¿Estás segura de que quieres ser mi amiga? ¡No! ¡No! ¡No te lo recomiendo! No, no, no, no, no. No me conoces. ¡Seguro mañana me dejarás! Sí, sí, mañana. ¡Estoy segura de que mañana te irás! ¡No! ¡Yo sé que lo harás! O tal vez no... Tal vez lo hagas hoy, más tarde, tal vez a las 6, después de verme comer tanto...
No, no. Todo está bien. No hay nada malo, para nada, ¿o sí? Tal vez... ¡No lo sé!
Es solo que... Tengo miedo, no entiendo nada, ¿debería seguir comiendo dulces? ¿Por qué no puedo dormir? ¡¿Por qué me sangran los dedos?! No entiendo... Debe haber algo malo conmigo, sí, eso.
¿Tú crees que hay solución? ¿Un camino? Pero, ¿de verdad existe? ¿Estás segura? Porque yo no... ¿Y si...? ¿Y si dejase de angustiarme? No, no puedo. La ansiedad es parte de mí.

Participant 3

Hola, ¿Cómo te llamas? ¿Cómo estás? Soy ansiedad y no dormí casi nada, despertaba en plena madrugada y no podía conciliar el sueño pues hoy iba a tener un día muy ocupado y no sabía cuáles serían mis impresiones y sentimientos. Los ruidos se hacen más fuertes y no puedo dormir, espero a que amanezca y cada vez me parece que el tiempo pasa más lento…

Participant 4

Ya estoy aquí. Calma, ya no estás sola. Puedes llamarme nadie, o quizá nada.
Discúlpame.
No estaré por mucho tiempo
Las noches despierta, conmigo la pasarás.
La migraña también seré yo.
Y ni que decir de tu mala alimentación
Discúlpame
No estaré por mucho tiempo

Theme: Depression

Similes

Participant 5

- Cuando estoy triste me siento como lavadora secando ropa.
- Como niño sin juguetes.

Participant 6

- Cuando estoy triste es como cuando una flor es arrancada.
- Cuando estoy triste es como cuando una piedra es tirada y se hunde en el mar.

Participant 7

- Cuando estoy triste me siento como un niño perdido en el supermercado.
- Cuando estoy triste me siento una melodía sin ritmo.

Participant 8

- Cuando estoy triste me siento como si fuera el único ser humano en la tierra.
- Cuando estoy triste me siento como si estuviera en un mar inmenso a punto de ahogarme

Participant 9

- Cuando estoy triste es como cuando una flor es arrancada
- Cuando estoy triste es como cuando una piedra es tirada y se hunde en el mar

Images

Participant 5

Not included due to copyright restrictions. The image was a graphic of a man with a tie shaking.

Participant 6

Not included due to copyright restrictions. The image was a graphic of the silhouette of a person, not from a visible gender, who has a hole in the chest.

Participant 7

Not included due to copyright restrictions. The image was a graphic of an empty, lit room, with blue walls, a light brown floor and two glass windows.

Participant 8

Not included due to copyright restrictions. The image was a photograph of a girl with a messy hairstyle sitting down on a chair, touching her cheeks with her hands and looking sad/disappointed at her bowl of food.

Participant 9

Not included due to copyright restrictions. The image was a graphic of a bird inside a cage with a few leaves floating on the outside.

Monologues

Participant 5

Hola me llamo ansiedad pero me puedes decir "ansi". Quiero decirte que siempre estaré ahí para ti. Pero no tengas miedo, ven, acércate, te voy dar un consejo. Cuando estés aburrido o estés cansado de hacer lo mismo, y no sabes qué hacer, corre y come todo lo que puedas. Sí, come cada vez que te sientas aburrido o no sabes qué hacer. No lo pienses más y come.
No hagas caso a los demás, ellos no saben nada. Están locos. Confía en mí y estarás de lo mejor, y ya no estarás aburrido o sin saber qué hacer. Solo ve y hazme caso. Eso sí, te estaré diciendo muchas cosas más y tú solo me tienes que escuchar a mí y a nadie más

Participant 6

Hola, me llamo vacío.
Cuéntame, ¿cómo te sientes hoy? Porque yo me siento como siempre. Me siento triste y eso me gusta, creo que es la mejor manera de vivir. Te sientes mucho mejor estando con dudas y preocupaciones.
Sí, yo mejor que nadie lo sé. Durante mucho tiempo he estado así. Creo que deberías intentarlo. Yo te puedo ayudar cuando lo desees, puedo ser tu amigo y cómplice para siempre. Sé lo que sientes, confía en mí, todo estará bien. Caminemos de la mano para iniciar tu nueva vida

Participant 7

Hola, me presento, soy el vació que sientes cuando no hay nadie contigo, ese vació que te hace dar cuenta que estás solo en este mundo y te hace ver que no eres necesario para nadie.
Aunque otros me identifican como soledad o confusión, ya que eso es lo que genero a los que me conocen... Así es, soy el amigo que necesitas para abrir los ojos y ver que esta vida, mejor dicho TU vida, no es importante en este mundo y no genera ningún cambio...
AJA! , veo que te genera recuerdos... Lástima que todos tienen un final malo y son provocados por ti, solo piénsalo un poco y te darás cuenta.
Bueno, un gusto poder hablar contigo... regresaré cuando menos te lo esperes... Más bien, te sugeriría que busques ayuda de alguien, ya que conmigo la necesitarás…

Participant 8

Hola me llamo depresión. Es de lo más genial estar triste y para seguir sintiéndote así, no debes comer, no debes dormir y mucho menos confiar en las personas. Créeme que eso te hará sentir bien

Participant 9

Un día cualquiera me habló mi sombra y me dice "Hola, me llamo Deón". Me quedé sorprendida y me dice "te haré sentir mucho dolor en tu pecho que no podrás soportar, y dolor psicológico. Y le digo "¿Por qué? ¿Por qué a mí?". Y me dice "Porque quiero ver si eres fuerte y si eres capaz de aguantar. Y le digo "¡Por favor, no! ¡No me hagas esto!". Comencé a gritar para que me escuche porque no me respondía.

Lifelines

Participant 1

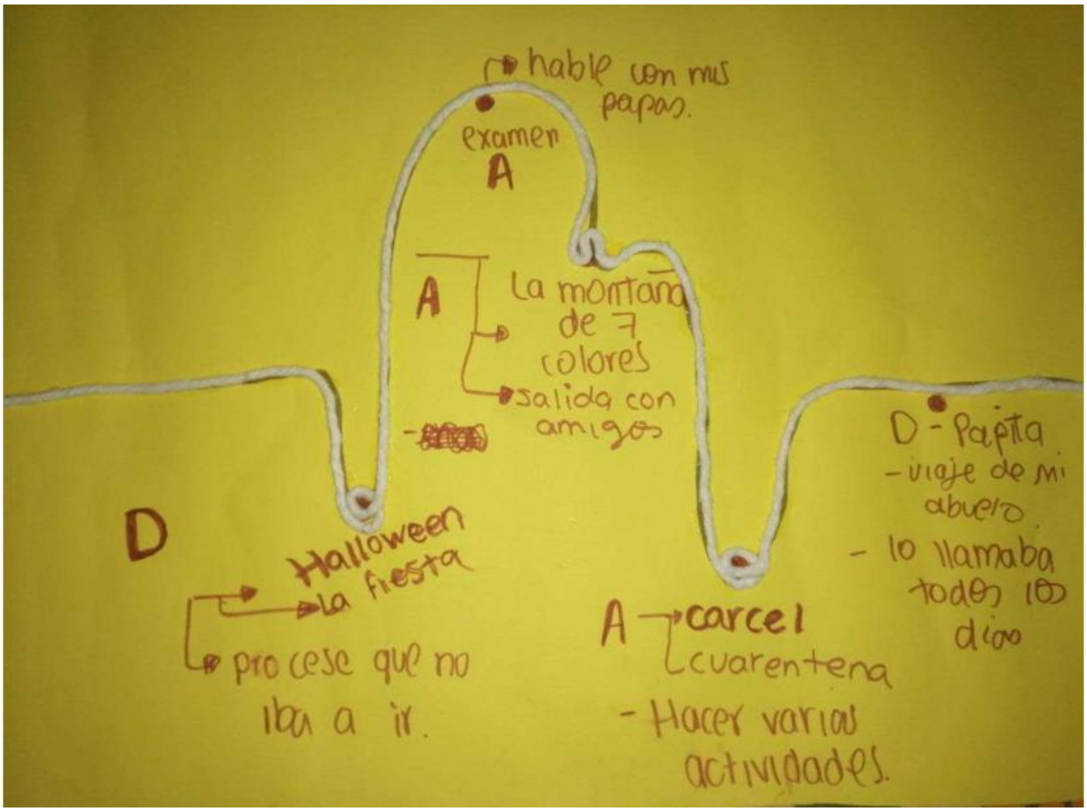

Participant 2

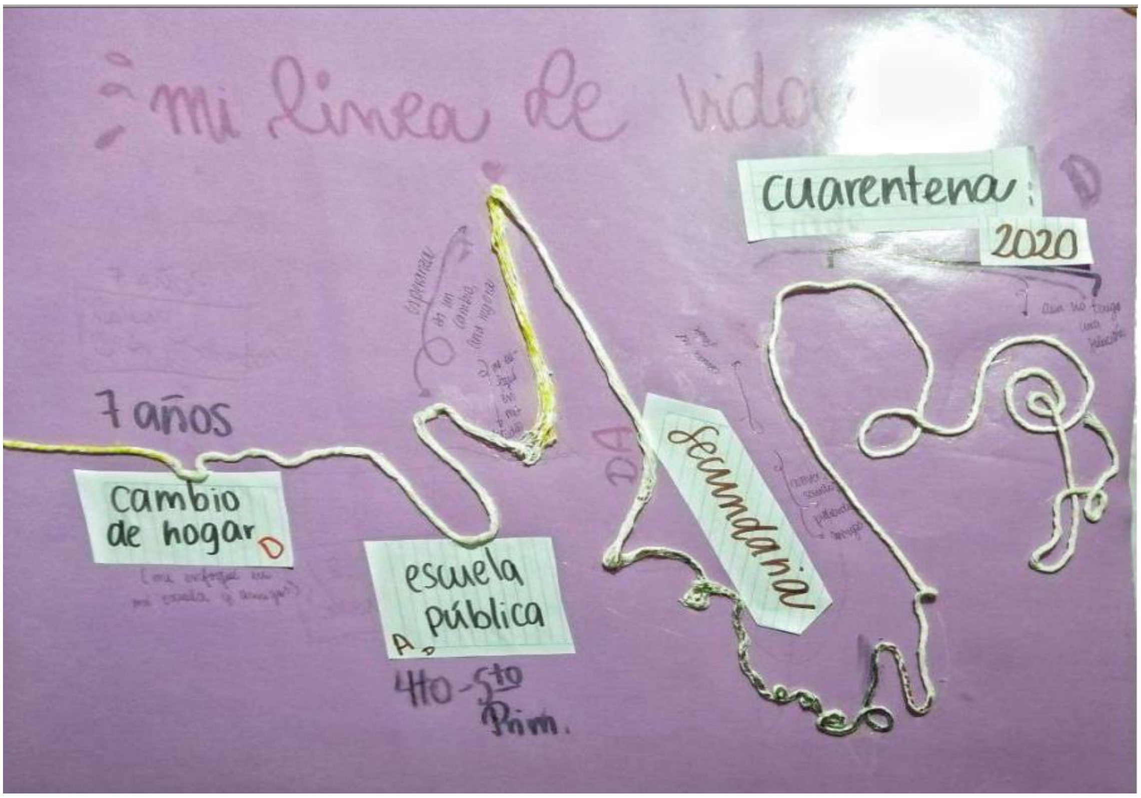

Participant 3

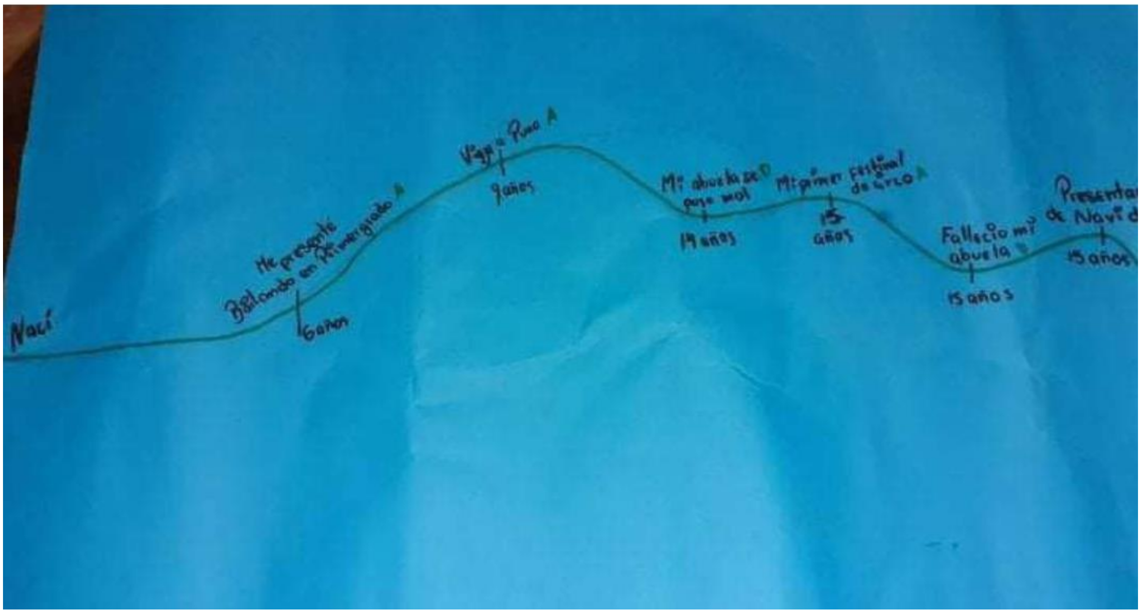

Participant 4

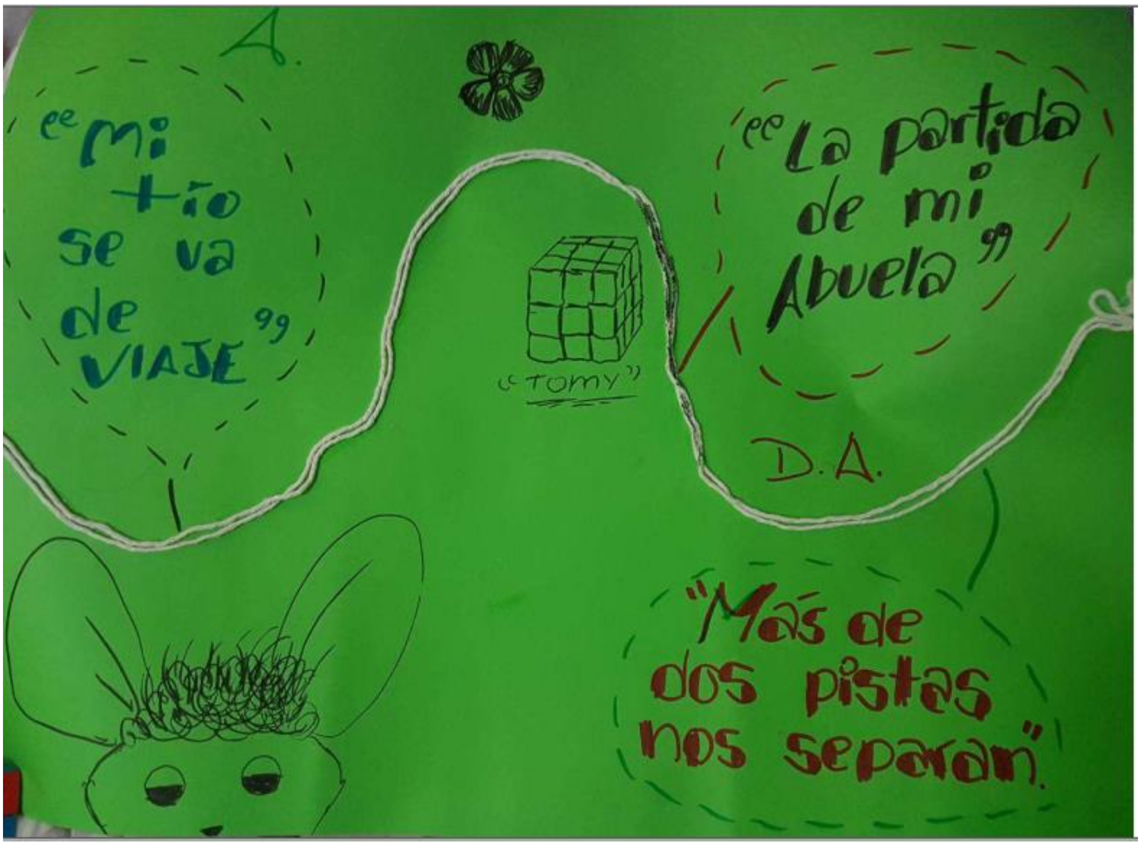

Participant 5

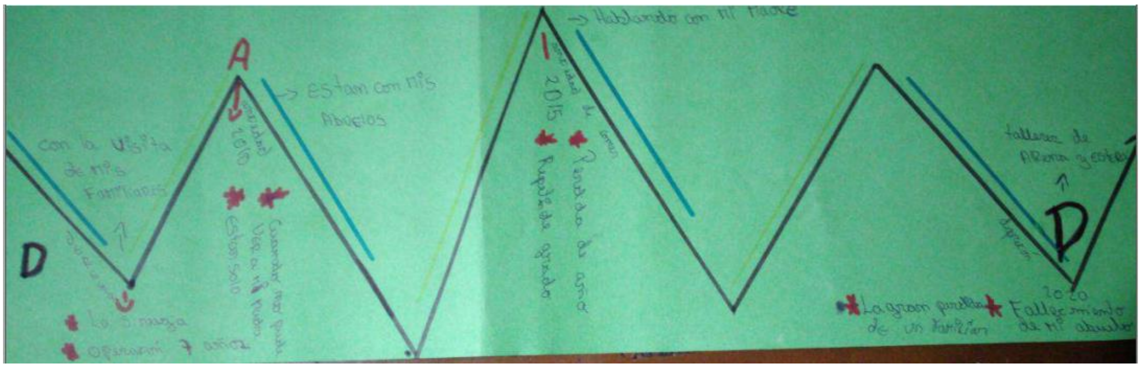

Participant 6

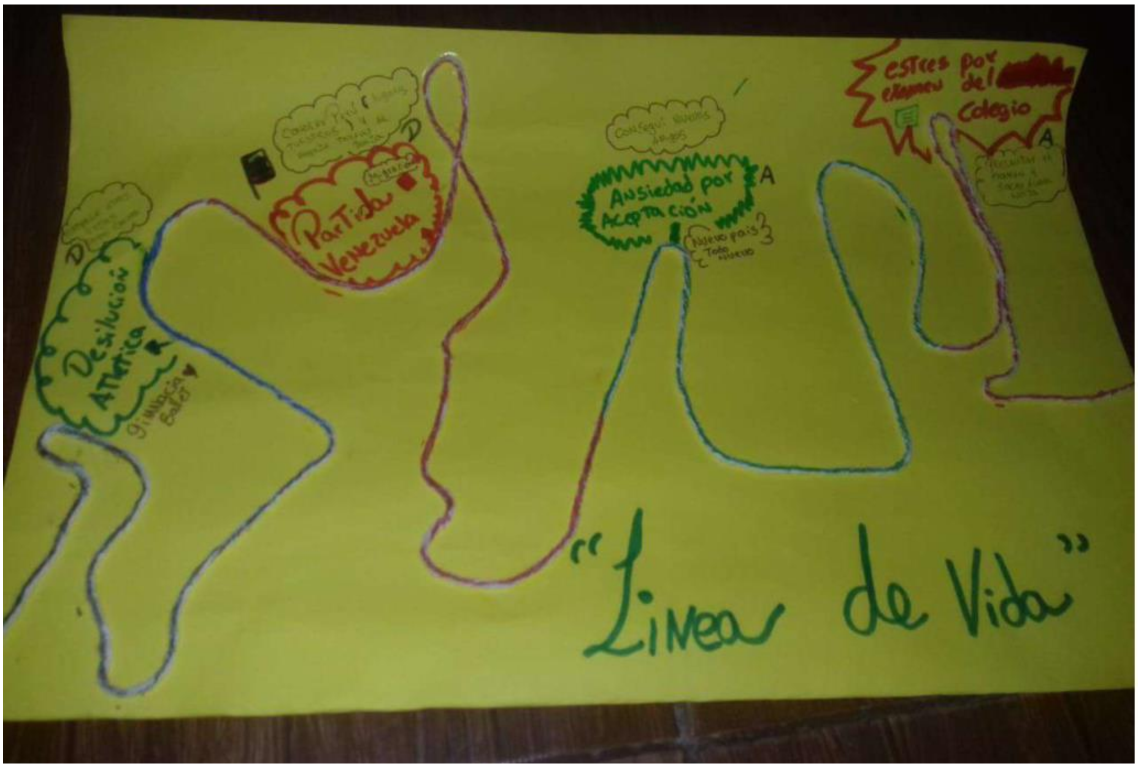

Participant 7

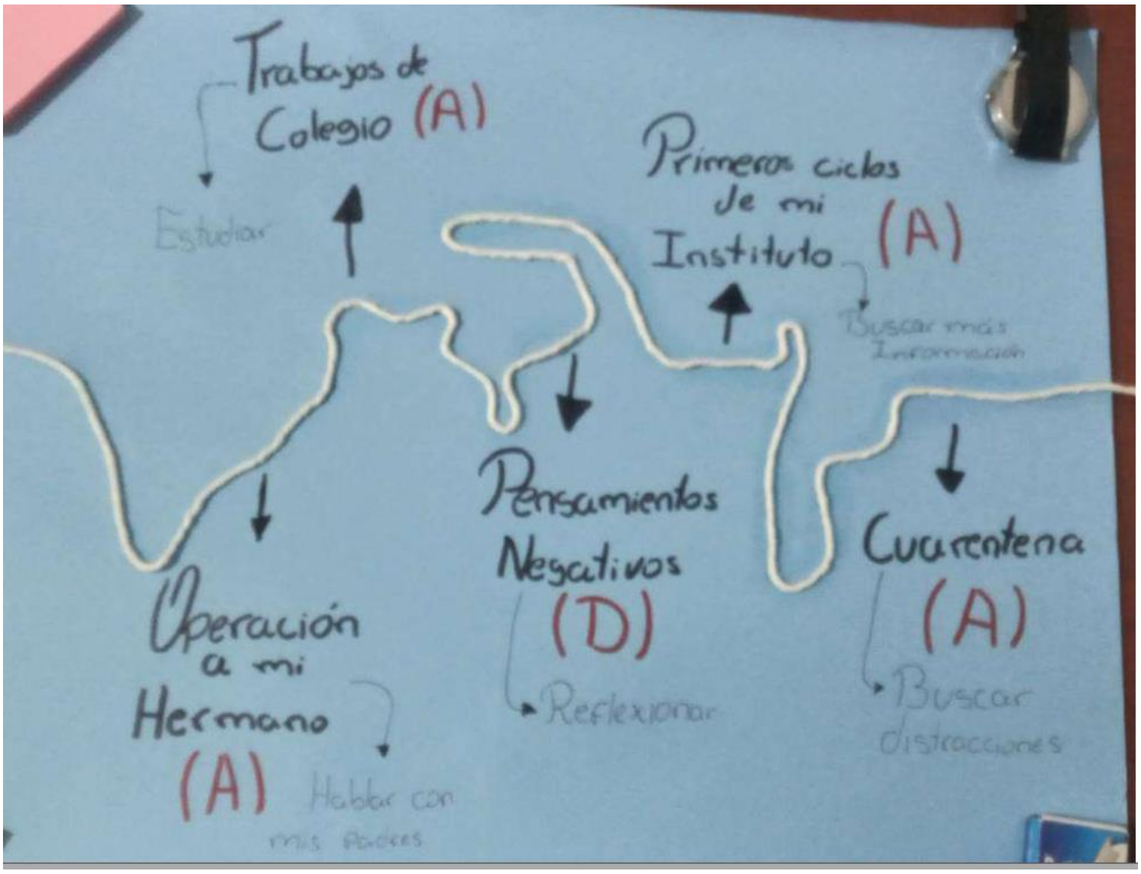

Participant 8

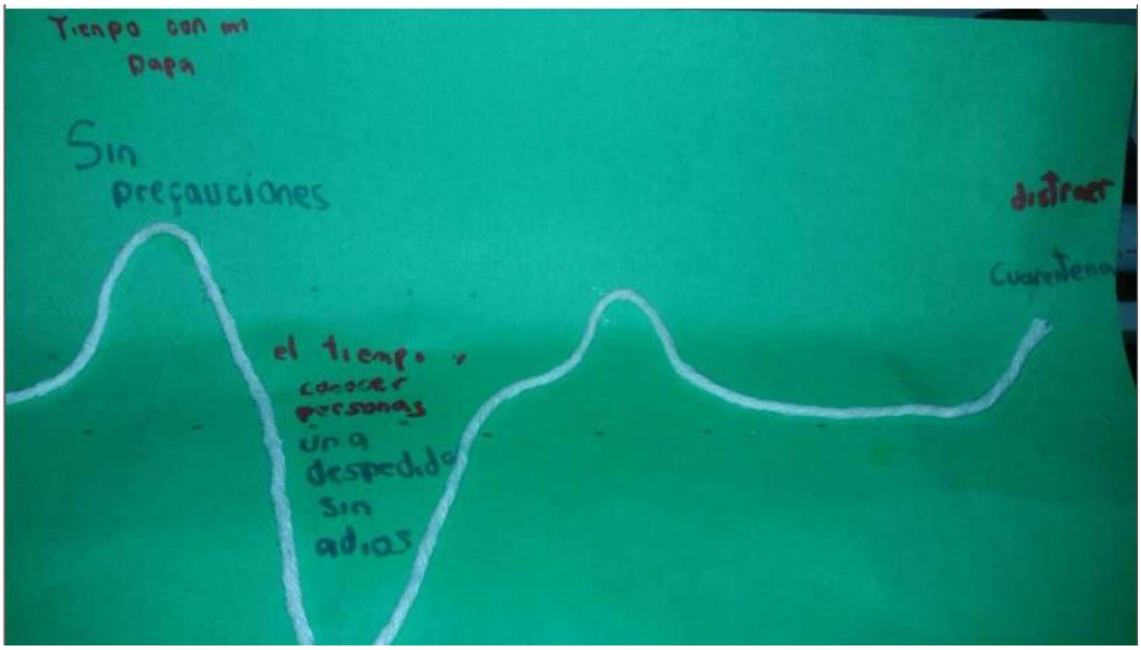

Participant 9

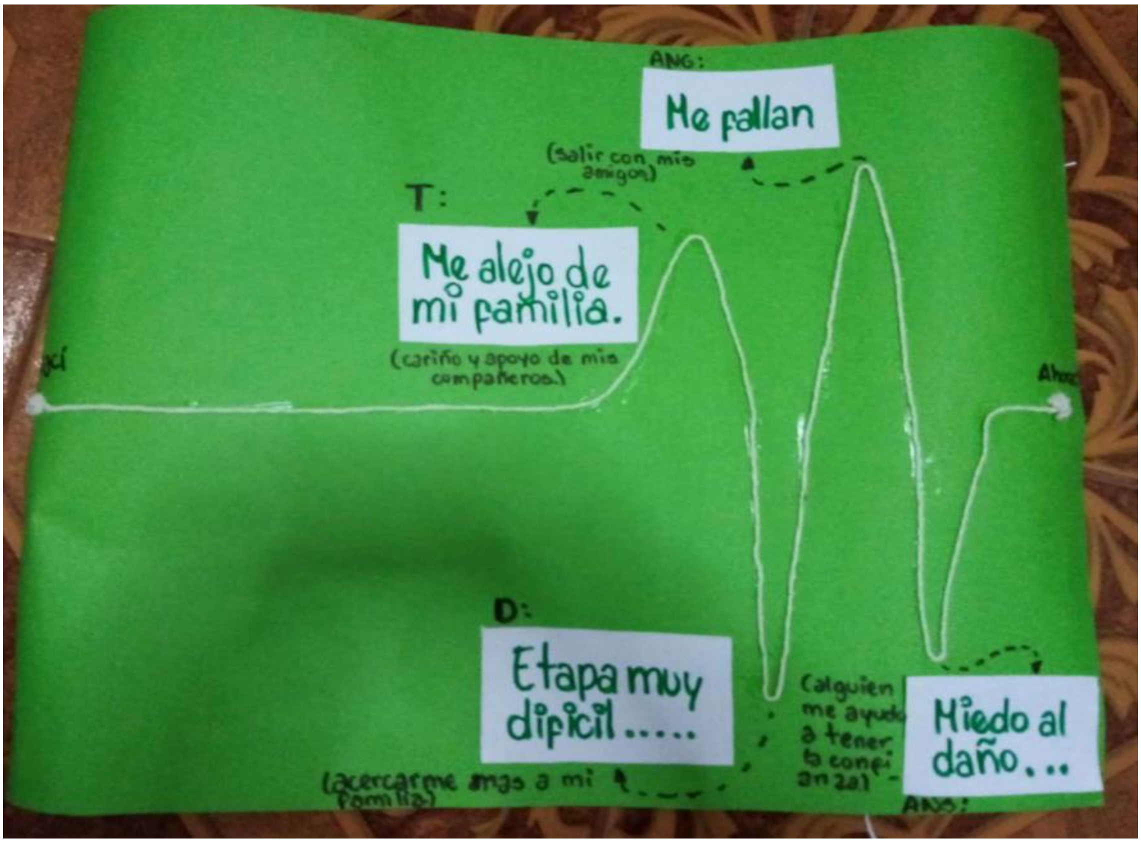

Supplement: Supplementary file 1 [file ijerph-19-15517-s001.zip › Supplementary File S1.pdf]
