# Supplementary material for: Using Arts-Based Methodologies to Understand Adolescent and Youth Manifestations, Representations, and Potential Causes of Depression and Anxiety in Low-Income Urban Settings in Peru
Source: Int J Environ Res Public Health. 2022 Nov 23;19(23):15517. doi: 10.3390/ijerph192315517 (PMC9737895; doi:10.3390/ijerph192315517)

Supplementary File S2

Artistic outputs of young adults

Theme: Anxiety

Similes

Participant 1

- Cuando estoy angustiado me siento como cronómetro a destiempo
- Cuando estoy angustiado me siento como pez a contracorriente
- Cuando estoy angustiado me siento como lluvia seca

Participant 2

- Cuando estoy angustiado me siento como un boxeador derrotado

Participant 3

- Cuando estoy angustiado me siento como barco anclado
- Cuando estoy angustiado me siento como soldado abandonado

Participant 4

- Cuando estoy angustiado me siento como el último palito de fósforo prendiendo la cocina
- Cuando estoy angustiado me siento como la cuerda de una guitarra eléctrica

Images

Participant 1

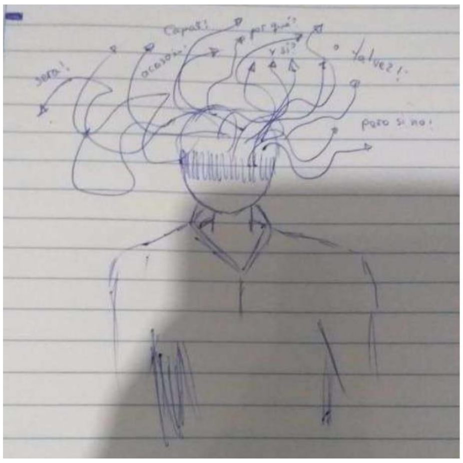

Participant 2

Not included due to copyright restrictions. The image is a photograph of an alone young man sitting on a bunch of stairs, seemingly from a stadium.

Participant 3

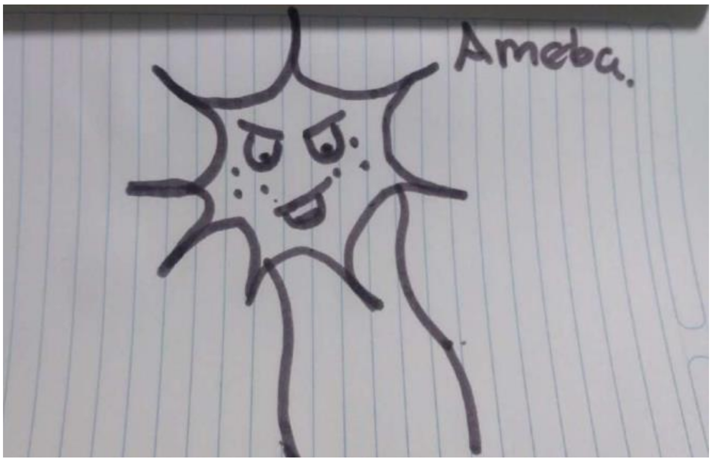

Participant 4

Not included due to copyright restrictions. The image is a graphic of a naked man looking backwards in a rigid position, with both legs and arms on a 45° degree angle in relation to the torso.

Monologues

Participant 1

Hola, soy… (titubea, no dice su nombre).
Mucho gusto. Quiero hacerles una pregunta. ¿Quieren contestarla? … Parece que no. Bueno, no importa.
Pero, saben, me gusta hacer… eso. Ya saben, lo que a todos les gusta. A ustedes, ¿no? … Podemos ser amigos, claro, si así quieren.
Hoy el día es muy bueno. Ojalá no pase nada malo, pero si lo pasa que no sea muy grande (con voz de miedo).
¿Usted cree que pase algo hoy? Yo creo que no. Podría ser como la vez que iba caminando y de la nada me caí. Alguien me vio. Bueno, no lo sé, pero me fui lo más rápido que pude. (Con voz un poco agitada)
Chao… un gusto conocerlos, mi nombre es… bueno, no importa.

Participant 2

¿No crees que es el momento de empezar algo más de lo que ya hicistes? Estuvistes informado de todas las guías den los foros pero aún así no tienes ese mínimo de valor a realizar un pequeño paso a que tu potencial se desperdicie en lo que te distrae. Sí, porque en realidad lo que te quita ese valioso tiempo… pues oye… terminará en solo una afición…

Participant 3

Hola, no vienen muy seguido por acá, ya los extrañaba. Sabía que no me abandonarían. ¿Por qué esas caras? No estén tristes. Les advertí que esas pastillas no les harían nada bien. Pasen, pueden comer todo lo que quieran. Qué importa el trabajo, el amor. Satisfagan su necesidad, no paren ahora que están conmigo. Yo los cuidaré. (Entre dientes) No los dejaré ir.

Participant 4

Soy la sombra que te acompaña siempre que al irte a la cama suelta un suspiro. La forma en la cual te alejas, el ser individuo cada vez más de la realidad, esta se deforma cambiándote. Como una persona íntima, abarco de manera tácita tus palabras. Soy tu ventana pero también la puerta y el piso. Las paredes. Tan presentes que se funden en un marco en el cual no existe relieve.

Theme: Depression

Similes

Participant 5

- Cuando estoy triste me siento como ciudad sin oxígeno
- Cuando estoy triste me siento como un árbol sin hojas
- Cuando estoy triste me siento como globo que se va volando
- Cuando estoy triste me siento como desierto sin agua
- Cuando estoy triste me siento como un mar sin peces
- Cuando estoy triste me siento como planta pisada
- Cuando estoy triste me siento como insecto muerto

Participant 6

- Cuando estoy triste me siento como un bolígrafo sin tinta (vacía y sola)
- Cuando estoy triste me siento como la sal en una barra de chocolate
- Cuando estoy triste me siento como un niña en un cuarto oscuro sola
- Cuando estoy triste me siento ahogada
- Cuando estoy triste me siento como si estuviera encerrada en un laberinto

Participant 7

- Cuando estoy triste me siento como un animalito que ha perdido su hábitat
- Cuando estoy triste me siento como una niña que no puede salir a jugar

Participant 8

- Cuando estoy triste me siento como una cajita de música descompuesta
- Cuando estoy triste me siento como la princesa Ariel ya que no tenía piernas para subir a la superficie

Images

Participant 5

Not included due to copyright restrictions. The image is a photograph of a woman and a man holding hands neat the beach, wearing autumn-like clothes.

Participant 6

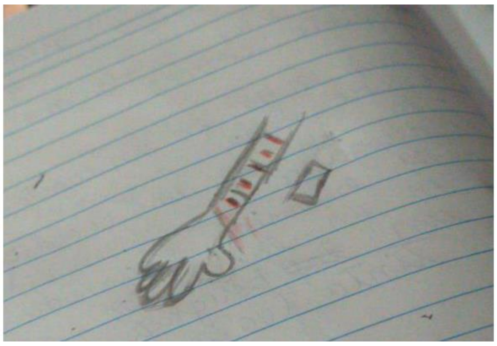

Participant 7

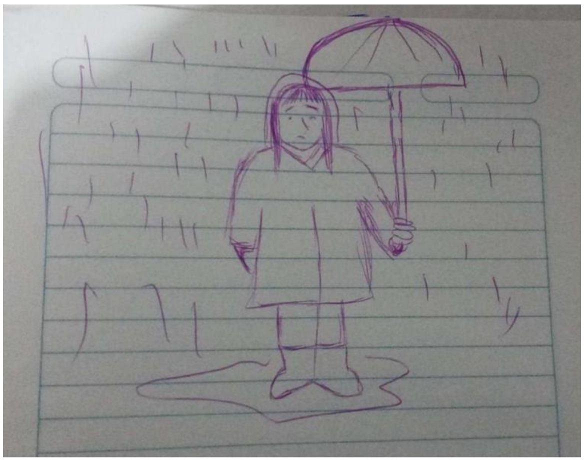

Participant 8

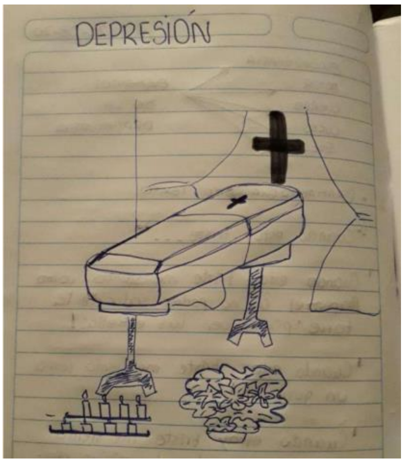

Monologues

Participant 5

¿Por qué no me miras de frente? ¿Acaso me sigues teniendo miedo? Sabes muy bien que te acompañé, estuve contigo sanando tu dolor. Además, si tú me lo pides, puedo volver a tomar tu mano y seguir caminando a tu lado.
¡Además! Si yo quisiera, estarías a mis pies, me debes mucho porque conmigo olvidaste tus penas. Cuando todos te dieron la espalda, te hice ser más fuerte, vencer tus miedos, buscar opciones para que puedas desahogar tu dolor y así me pagas queriendo irte de mi lado.

Participant 6

Hoy, como de costumbre, llegué al colegio y, como de costumbre… ella me comenzó a hablar. No como antes, sino con un tono amable y palabras hirientes que de nuevo me lleva a esto que no me causa dolor, algo de satisfacción. Lo que ella dice, no sé si sea verdad, pero siento que… sí. No valgo nada.
Eso me hace creer. Al llegar a casa me cubro el brazo con la casaca, saludo a mi mamá y ni me responde. Al rato me llama y me dice "¡bastardo! ¡qué has estado

haciendo! ¡vagando paras!" Cuando en verdad estaba arreglando su cuarto, pero claro, como no nací en el matrimonio, ella me llama como quiere. En verdad ¿soy alguien? ¿En verdad valgo?

Participant 7

Hola, he llegado hasta ti. Tu tristeza me ha llamado, tu discusión con la felicidad me hace fuerte. Pero no te preocupes, yo estoy aquí para acobijarte. ¿Tú sabes quién soy? Sí, soy la depresión. Sé que te sientes cansada, sin ánimos y puede que pienses que el mundo se va a acabar. Pero, ven, estamos para acompañarnos.
Esto puede durar unos días, tal vez meses o años. Tú, ¿quieres saber cómo termina esta etapa? Pues, yo estaré aquí hasta que tú misma te des cuenta y recuperes tu fuerza, ganas y valentía. Mientras… acompañaré tu lento caminar.

Participant 8

Bueno, al parecer estuviste un buen tiempo sin mí. Lo último que te ocurrió es horrible, triste y claro, yo, deprimente, pero no te preocupes, estoy para acompañarte. Cuando tiempo tú me lo permitas.

Lifelines

Participant 1

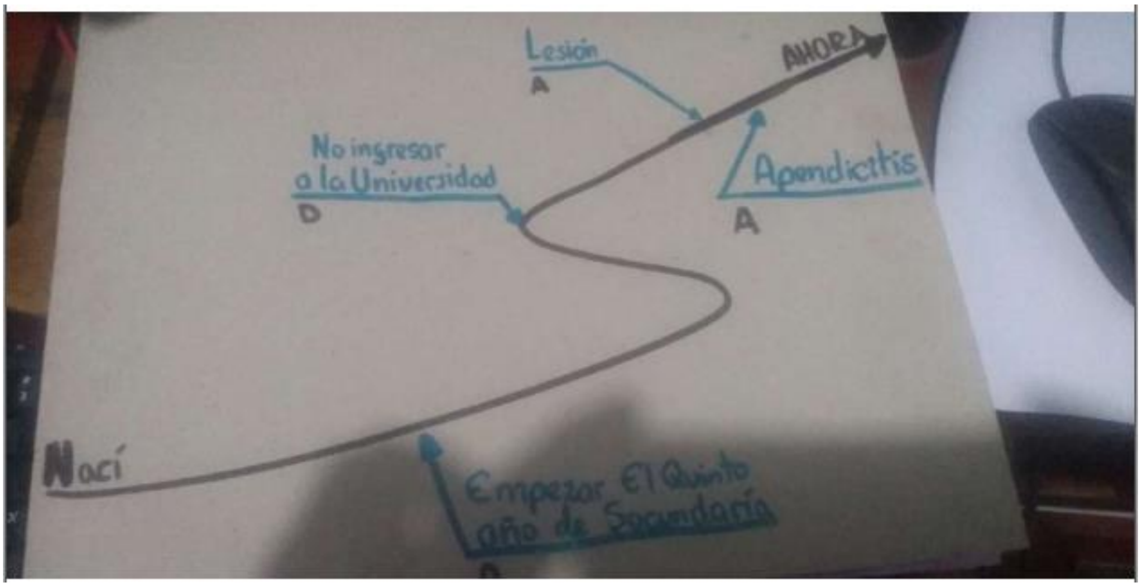

Participant 2

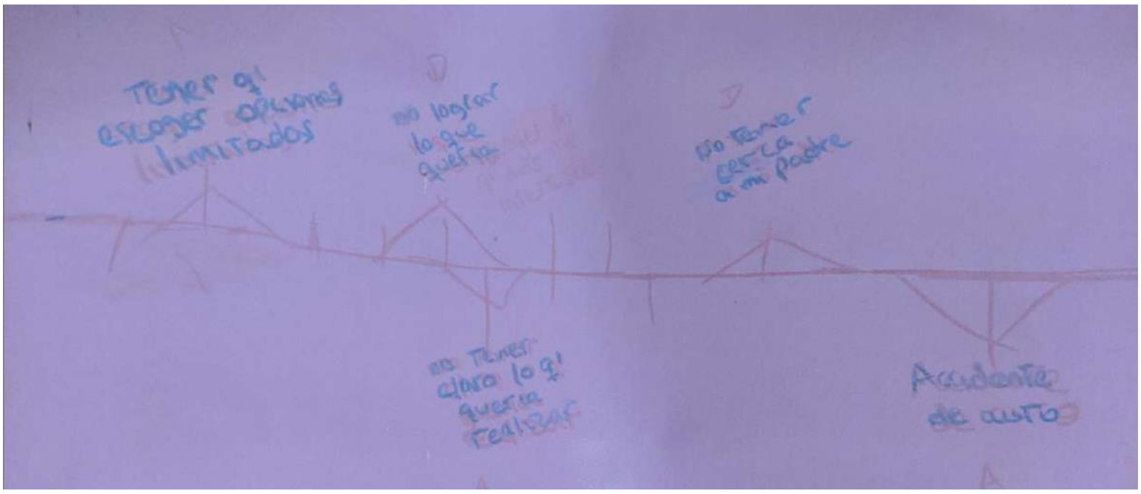

Participant 3

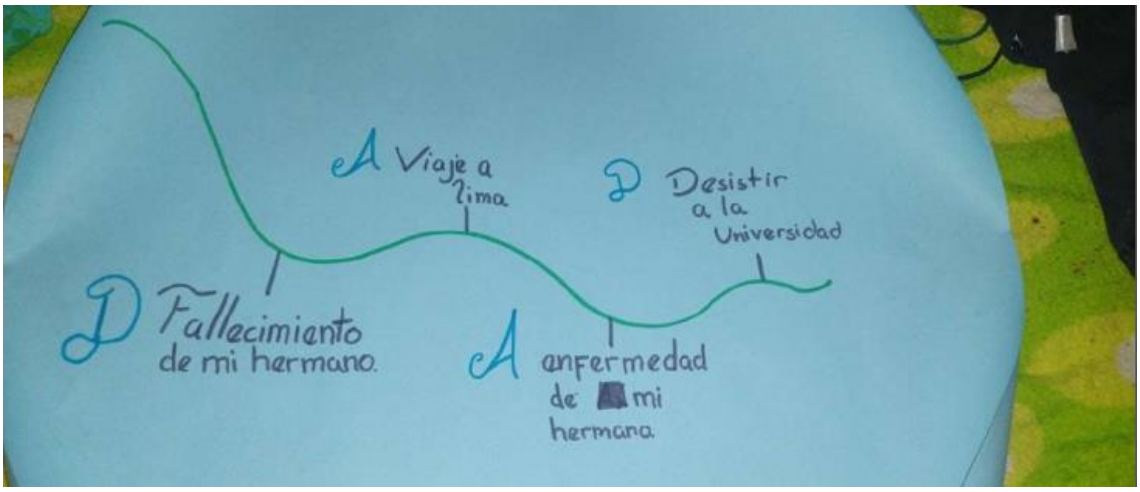

Participant 4

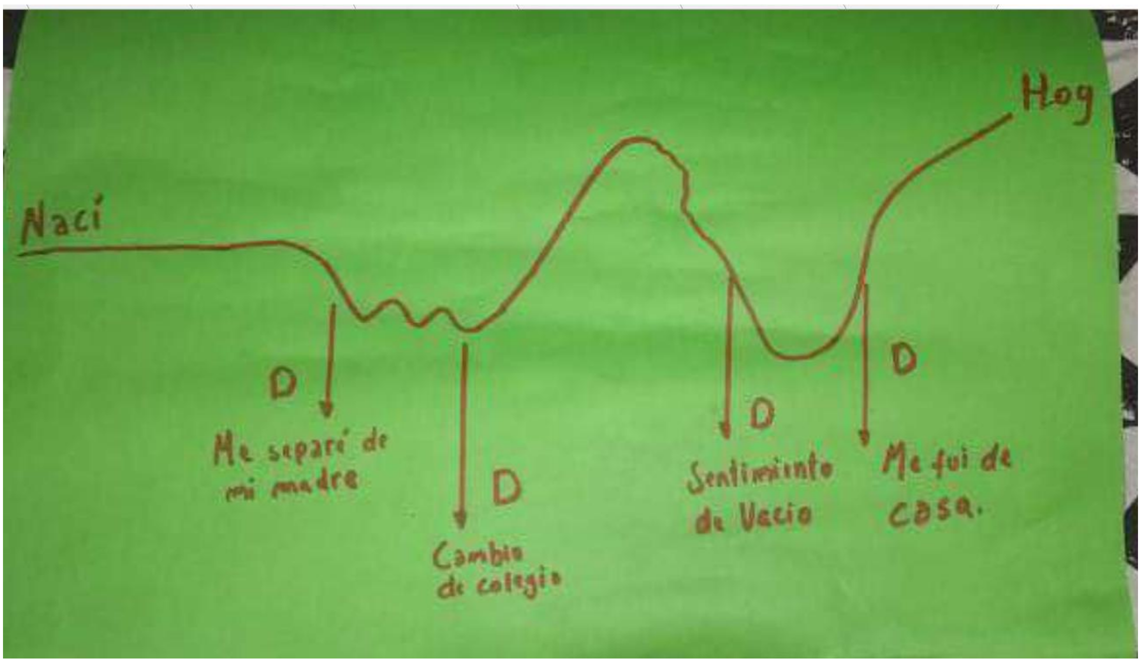

Participant 5

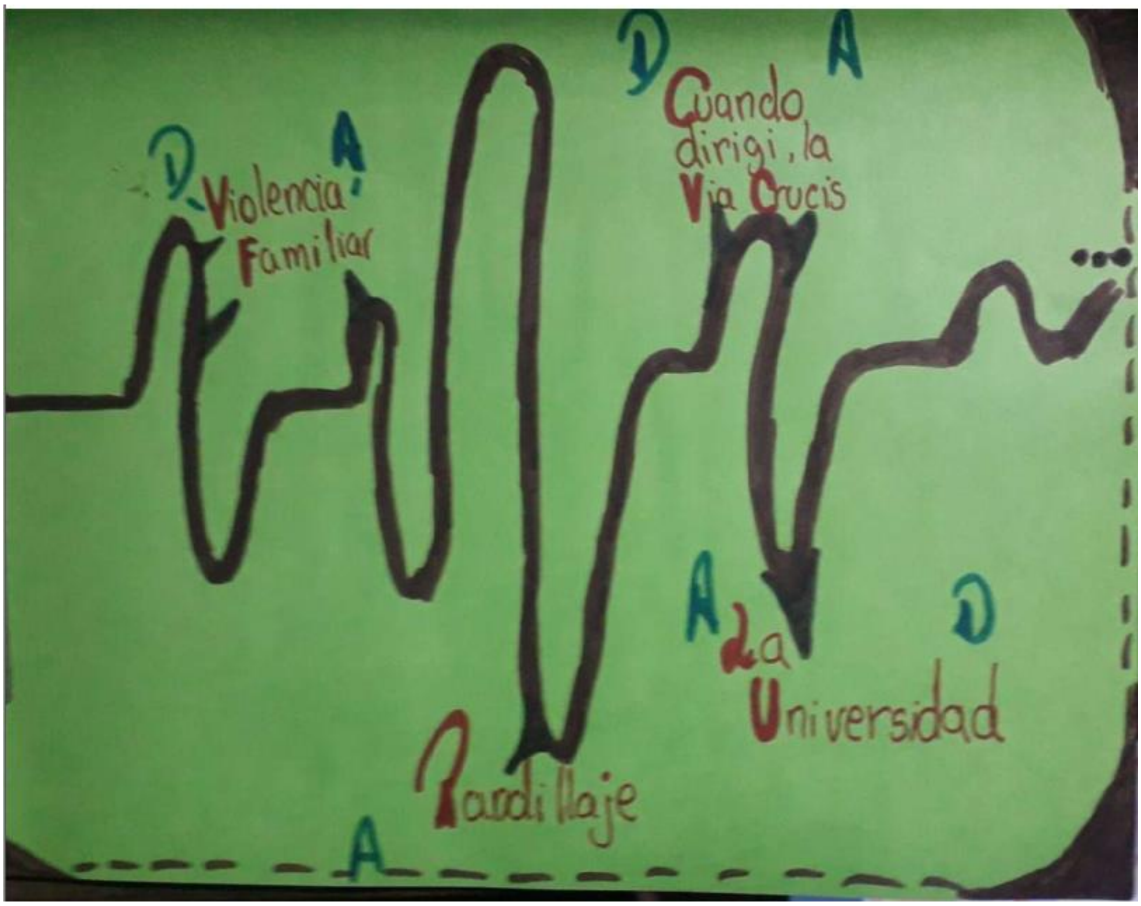

Participant 6

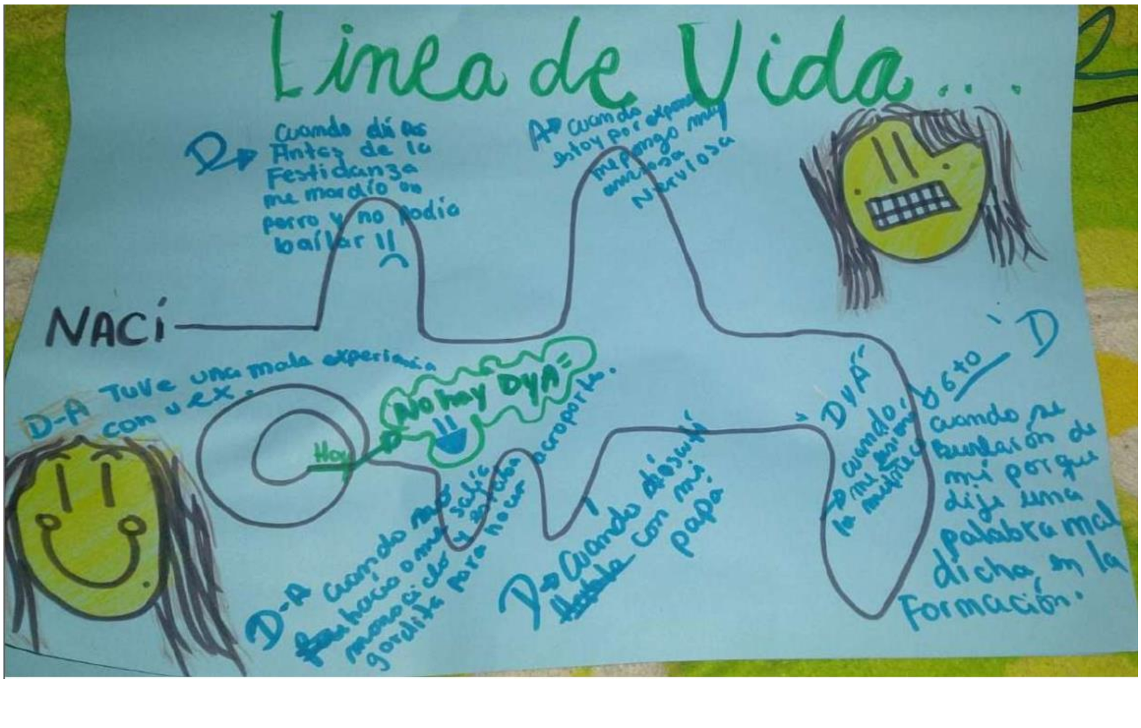

Participant 7

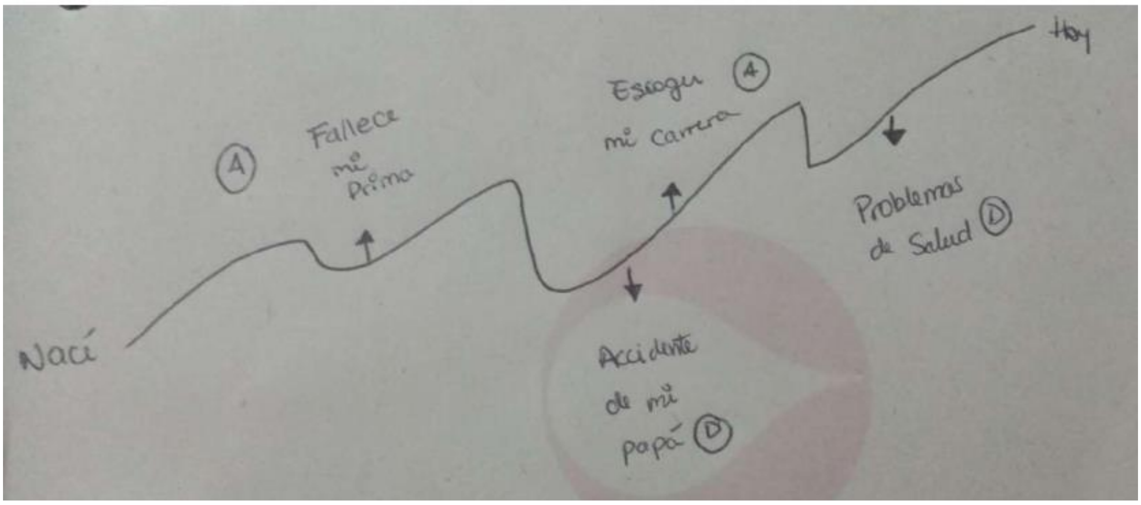

Participant 8

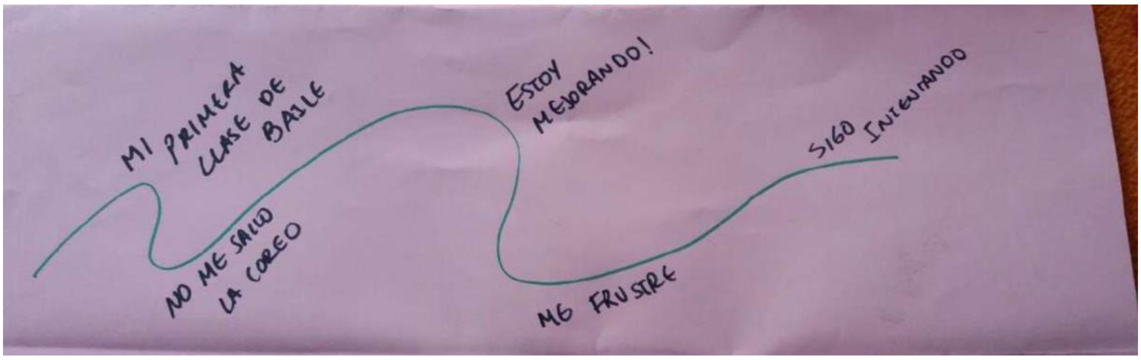

Supplement: Supplementary file 1 [file ijerph-19-15517-s001.zip › Supplementary File S2.pdf]
